# Managing pregnancy in a spinal muscular atrophy type III patient in Indonesia: a case report

**DOI:** 10.1186/s13256-021-03226-1

**Published:** 2022-01-16

**Authors:** Cempaka Thursina Srie Setyaningrum, Indra Sari Kusuma Harahap, Dian Kesumapramudya Nurputra, Irwan Taufiqur Rachman, Nur Imma Fatimah Harahap

**Affiliations:** 1 Department of Neurology, Faculty of Medicine, Public Health and Nursing, Universitas Gadjah Mada, Yogyakarta, Indonesia; 2 Department of Pediatrics, Faculty of Medicine, Public Health and Nursing, Universitas Gadjah Mada, Yogyakarta, Indonesia; 3 Department of Obstetric and Gynecology, Faculty of Medicine, Public Health and Nursing, Universitas Gadjah Mada, Yogyakarta, Indonesia; 4 Department of Clinical Pathology, Faculty of Medicine, Public Health and Nursing, Universitas Gadjah Mada, Yogyakarta, Indonesia

**Keywords:** Case report, Spinal muscular atrophy, Pregnancy, Yogyakarta, Indonesia

## Abstract

**Background:**

Spinal muscular atrophy is a genetic disorder characterized by degeneration of lower motor neurons, leading to progressive muscular atrophy and even paralysis. Spinal muscular atrophy usually associated with a defect of the *survival motor neuron 1* (*SMN-1*) gene. Classification of spinal muscular atrophy is based on the age of onset and maximum motor function milestone achieved. Although spinal muscular atrophy can be screened for in newborns, and even confirmed earlier genetically, this remains difficult in Third World countries such as Indonesia.

**Case presentation:**

A 28-year-old Asian woman in the first trimester of her second pregnancy, was referred to the neurology department from the obstetric department. Her milestone history showed she was developmentally delayed and the ability to walk independently was reached at 26 months old. At 8 years old, she started to stumble and lose balance while walking. At this age, spinal muscular atrophy was suspected because of her clinical presentations, without any molecular genetic testing. She was married at the age of 25 years and was soon pregnant with her first child. At the gestational age of 32 weeks, her first pregnancy was ended by an emergency caesarean section because of premature rupture of the membranes. In this second pregnancy, she was referred early to the general hospital from the district hospital to receive multidisciplinary care. She and her first daughter underwent genetic testing for spinal muscular atrophy, which has been readily available in our institution since 2018, to confirm the diagnosis and prepare for genetic counseling.

**Conclusions:**

Managing pregnancy in a patient with spinal muscular atrophy should be performed collaboratively. In this case, genetic testing of spinal muscular atrophy and the collaborative management of this patient allowed the clinical decision making and genetic counseling throughout her pregnancy and delivery.

## Background

Spinal muscular atrophy (SMA) is a genetic disorder characterized by degeneration of lower motor neurons, leading to progressive muscular atrophy, muscle weakness, and muscle paralysis. SMA is usually associated with defect of the *survival motor neuron-1* (*SMN-1*) gene, localized in 5q11.2–q13.3 [1, 2]. Classification of SMA is based on the age of onset and maximum motor function milestone achieved: SMA type I (Werdnig–Hoffmann disease) has onset in the first month of life, with a disability to sit unsupported and needing breathing support, with a survival chance of only up to 2-years old; SMA type II has onset between 6 and 18-months old, with the best motor ability to sit alone without support; SMA type III (Kugelberg–Welander disease) has onset between 18 months and 30-years old, with the ability to stand and walk without support; and SMA type IV has adulthood onset and tends to have normal mobility with mild muscle weakness [3, 4].

The estimated incidence of SMA is 1 in 10,000 newborns and the prevalence is 1–2 people per 100,000, with an overall carrier frequency of 1 in 54 in the general population. SMA type I is believed to have the highest incidence and mortality rate in newborns and early childhood, but SMA type III has a wide range in age of onset that may cause variations in its clinical manifestations [3, 5]. There is currently no national official report on the incidence and prevalence of SMA in Indonesia. This may be due to the lack of availability in genetic testing in Indonesia. Thus, an established definitive diagnosis of SMA cannot be achieved. The lack of information regarding SMA in Indonesia makes further study necessary. Here we report an Indonesian woman with SMA type III who successfully delivered two healthy children despite not receiving specific treatment for her progressive muscle weakness.

## Case presentation

A 28-year-old Asian woman in the first trimester of her second pregnancy was referred to the neurology department from the obstetric department for multidisciplinary antenatal care regarding her muscle weakness. Her milestone history showed she was born prematurely at 32 weeks gestational age, with low birth weight of 1800 g. She was developmentally delayed and the ability to walk independently was reached at the age of 26 months old. At the age of 8 years, she started to stumble and lose balance while walking. She had no family history of hereditary muscle weakness. At this age, SMA was suspected because of her clinical presentations, without any molecular genetic testing. She underwent physical and occupational therapy in a district hospital for 1 year, but her parents decided to stop the therapy due to financial problems. There was no universal national health insurance coverage in Indonesia in the early 2000s. It was quite troublesome for a family with a disadvantaged child who lived in the rural area to visit a district hospital located in the downtown of the city. At the age of 12 years, she could no longer walk on her own, started ambulating using a wheelchair, and stopped going to the formal school.

Despite her progressive muscle weakness, the patient could be reproductively active. She was married at the age of 25 years and was soon pregnant with her first child. She went to a district hospital for antenatal care. At 32 weeks gestation, she had an emergency caesarean section because of premature rupture of the membranes. The baby girl was born prematurely with a birth weight of 2600 g. She continued to grow healthily to date without any observed muscle weakness.

In this second pregnancy, she and her first daughter underwent genetic testing for SMA, which has been readily available in our institution since 2018, to confirm the diagnosis and prepare for genetic counseling. The results showed homozygous deletion of *SMN1* exons 7 and 8 in the patient, but not her daughter, confirming that the mother and daughter were patient and carrier, respectively. However, copy number analyses and prenatal diagnostic testing for SMA were not readily available in our hospital. The patient and her family underwent genetic counseling in which they were determined to continue the pregnancy. No particular concern was raised regarding the fetal development in utero until the third trimester. She was planned for a scheduled caesarean section at 38 weeks of gestational age. This was done considering that at this gestational age, fetal respiratory insufficiency and prematurity could be prevented.

### Examinations/investigations

**Fig. 1:**
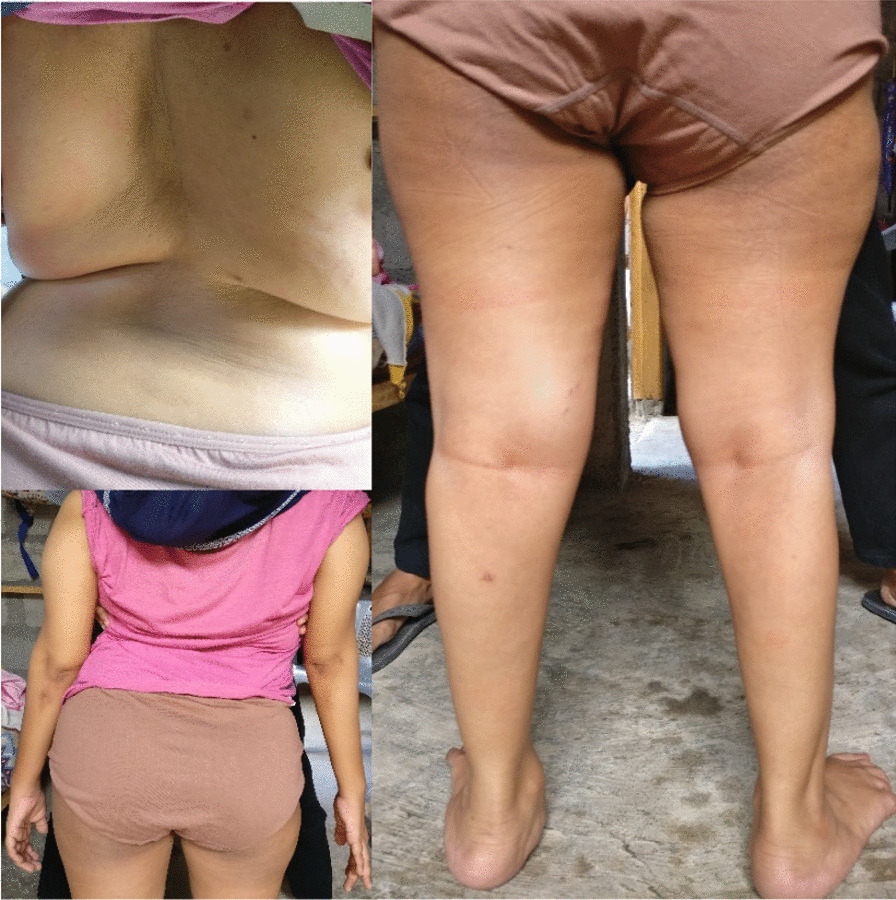
Patient’s clinical condition. This shows the current condition of the patient, with severe scoliosis and decreased muscle tone in upper and lower extremities. In the pictures, the patient was supported by her husband who stood in front of her and held her body on both sides because she could not stand independently. This picture has the patient’s permission for publication and research purpose

In the neurology clinic, the patient presented in a wheelchair with severe scoliosis and disability to walk or stand independently. There was evidence of flaccid weakness in all her limbs, particularly the lower ones, with decreased muscle tone, trophy, and strength. Muscle weakness was more prominent in the proximal parts of her lower limbs (Fig. 1).

**Fig. 2:**
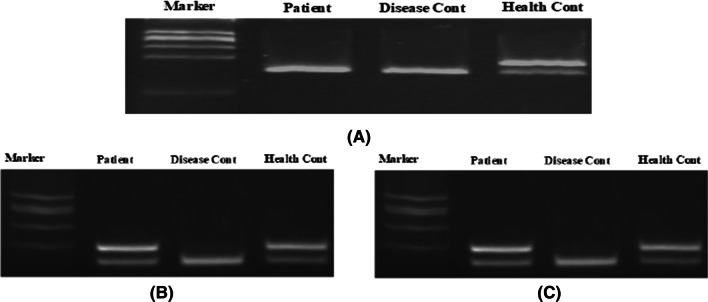
The genetic results (*SMN-1*) from exon 7 from patient and her children. **A** The patient’s genetic result show that she had deletion in *SMN-1*. **B** Genetic result of patient’s first daughter showed no deletion in *SMN-1*. **C** Genetic result of patient’s second son showed no deletion in *SMN-1*. This picture has the patient’s permission for publication and research purpose

Electroneuromyography showed an axonal neuropathy of the spinal nerve roots innervating the lower limbs and bilateral median nerves axonopathies without cervical radiculopathy. Electromyography showed a chronic denervation without reinnervation. Molecular genetic testing for SMA showed a homozygous deletion of *SMN1* exons 7 and 8, and retention of *SMN2* exons 7 and 8, as well as NLR family apoptosis inhibitory protein (*NAIP*) exon 5 (Fig. 2)

### Treatments/intervention

Despite the current successful treatment of SMA using antisense oligonucleotide Nusinersen and gene replacement therapy, none of these drugs were available in Indonesia. Therefore, the patient did not receive specific medication for her muscle weakness condition. She did not continue her physical and occupational therapy. At first, this was due to the lack of universal health coverage, but currently it was due to her difficulty in managing her time and lack of readiness to pay a visit to the hospital.

### Outcomes/follow-up

The patient’s second child was delivered at 38 weeks gestational age. The elective caesarean section was followed by Pomeroy sterilization on indications of a mother with SMA, history of previous caesarean section in the last 3 years, and having enough children. The second child was male with a birth weight and length of 2932 g and 42 cm, respectively. No adverse event was found in this procedure, either for the mother or the baby. Genetic testing for SMA was performed on the newborn soon after birth The results showed the retention of *SMN1* exons 7 and 8, indicating that this baby was a carrier. Until this case report was made, there were no reports on the patient’s children that suggested any muscle weakness concerning SMA or other neuromuscular disorders

## Discussion and conclusions

Neuromuscular diseases, either hereditary or nonhereditary, require further workups with advanced resources, such as neurophysiologic testing, muscle biopsies, tissue damage markers, and molecular genetic tests. In Indonesia, these examinations could only be performed in a referral hospital located in the capital city of a province. In this case, diagnosis of spinal muscular atrophy (SMA) type III was made based on the onset of the symptoms and clinical neurological examinations, and confirmed by genetic examinations for SMA. This case also described a patient with SMA type III who managed to survive, have a reproductively active life, and gave birth to two healthy children (carriers) without any specific medications for her condition. In Indonesia, it is difficult to diagnose SMA by genetic testing since the test is not widely available. To our knowledge, our institution established the first genetic testing for SMA in Indonesia using qualitative polymerase chain reaction-restriction fragment length polymorphism (PCR-RFLP) analysis. However, this basic genetic testing is still not widely used for the diagnosis SMA since it is not covered by the national health insurance. More advanced and complicated genetic tests, such as copy number analysis, sequencing, and prenatal genetic testing, were not available in our institution. Taking these circumstances into account, it would be difficult to establish a definitive diagnosis of SMA in Indonesia.

This case was also the first collaborative case with a multidisciplinary approach between the obstetric, neurology, pediatric, and anesthesiology departments in our hospital. Cases and studies of SMA patients in pregnancy have been reported several times. There were three reports of SMA patients that we studied for the preparation of the patient’s pregnancy and obstetric management. The first study was conducted by Carter *et al.* in 1994, and reported two cases of SMA type II women who successfully delivered healthy babies by vaginal delivery and caesarean section, each at 39 weeks gestational age [6]. The second study in 2009 by Flunt *et al.*, reported a high-risk pregnancy in a 32-year-old woman with severe SMA type II. This pregnancy was ended by a planned caesarean section at 28 weeks owing to maternal and fetal conditions of prematurity, kyphoscoliosis, anticipated cephalopelvic disproportion, and primiparity. Twenty-eight weeks gestation was chosen to end the pregnancy after weighing the best balance of fetal respiratory maturity and maternal anthropometry [7]. The third case was reported by Howarth in 2011 regarding a type III SMA patient who underwent a caesarean section at 32 weeks gestation. This delivery was planned because of increasing abdominal and back pain, particularly after 30 weeks, from maternal factors. For the fetal factors, it was an anticipated premature delivery considering the prematurity of her respiratory tracts. By referring to these case reports, our team considered preserving the pregnancy as long as possible to prevent prematurity [8]. We closely monitored any discomfort that may appear from the maternal condition due to her deformities, and from the fetal condition for any signs of fetal growth retardation. Fortunately, no maternal and fetal factors appeared until 38 weeks when the caesarean section was planned for delivery.

Pregnancy in SMA patients should be treated comprehensively and managed carefully. Maternal factors such as muscle weakness, deformities, and restrictive ventilatory pattern (lung capacity and compliance reductions), as well as social issues such as stigma and concerns from family and environment may arise [8, 9]. This pregnancy can be categorized as a high-risk pregnancy since it may lead to complications such as the possibility of preterm labor, miscarriage, intrauterine growth restriction (IUGR), and maternal back pain due to increasing pressure from abdominal enlargement [9]. These complicated conditions required good teamwork from a multidisciplinary team of healthcare professionals to maximize the outcomes, both for the mother and her baby [7]. The ideal team should include an obstetrician, neurologist, neonatologist, geneticist, anesthesiologist, and respiratory specialist.

Dealing with pregnancy in a patient with SMA is not easy, clinically or socially. However, this report shows that a patient with SMA could have a normal reproductive life while having a marriage and healthy children. Although this is not a common case that will be encountered in routine clinical practice, managing pregnancy in a patient with SMA should be performed collaboratively. By taking every maternal and fetal factor into account, a successful pregnancy and delivery in an SMA patient could be managed well.

A hereditary chronic progressive muscular atrophy, such as spinal muscular atrophy, might be suspected in a patient who met the clinical presentation of this disease despite the lack of family history and no available genetic testing. Multidisciplinary care should be maintained beginning with the antenatal care in treating pregnancy in a woman with spinal muscular atrophy. Early detection of SMA and genetic counseling should be given to the family, even though this case is rarely seen in daily clinical practice.

## Data Availability

Any statement, information, and data in this case report was already obtained an approval and acceptance from the subjects and their family.

## Abbreviations

SMA: Spinal muscular atrophy
SMN-1: Survival motor neuron-1
SMN-2: Survival motor neuron-2
NAIP: NLR family apoptosis inhibitory protein
PCR-RLFP: Polymerase chain reaction-restriction fragment length polymorphism
IUGR: Intrauterine growth restriction
